# Molecular identification for epigallocatechin-3-gallate-mediated antioxidant intervention on the H_2_O_2_-induced oxidative stress in H9c2 rat cardiomyoblasts

**DOI:** 10.1186/1423-0127-21-56

**Published:** 2014-06-09

**Authors:** Wei-Cheng Chen, Shih-Rong Hsieh, Chun-Hwei Chiu, Ban-Dar Hsu, Ying-Ming Liou

**Affiliations:** 1 Institute of Bioinformatics and Structural Biology, National Tsing Hua University, Hsinchu 30013, Taiwan; 2 Department of Cardiovascular Surgery, Taichung Veterans General Hospital, Taichung 407, Taiwan; 3 Department of Life Sciences, National Chung Hsing University, Taichung 40227, Taiwan; 4 Rong Hsing Research Center for Translational Medicine, National Chung Hsing University, Taichung 40227, Taiwan

**Keywords:** EGCG, H9c2, Oxidative stress, Proteomics analysis, Survival pathway

## Abstract

**Background:**

Epigallocatechin-3-gallate (EGCG) has been documented for its beneficial effects protecting oxidative stress to cardiac cells. Previously, we have shown the EGCG-mediated cardiac protection by attenuating reactive oxygen species and cytosolic Ca^2+^ in cardiac cells during oxidative stress and myocardial ischemia. Here, we aimed to seek a deeper elucidation of the molecular anti-oxidative capabilities of EGCG in an H_2_O_2_-induced oxidative stress model of myocardial ischemia injury using H9c2 rat cardiomyoblasts.

**Results:**

Proteomics analysis was used to determine the differential expression of proteins in H9c2 cells cultured in the conditions of control, 400 μM H_2_O_2_ exposure for 30 min with and/or without 10 to 20 μM EGCG pre-treatment. In this model, eight proteins associated with energy metabolism, mitochondrial electron transfer, redox regulation, signal transduction, and RNA binding were identified to take part in EGCG-ameliorating H_2_O_2_-induced injury in H9c2 cells. H_2_O_2_ exposure increased oxidative stress evidenced by increases in reactive oxygen species and cytosolic Ca^2+^ overload, increases in glycolytic protein, *α-enolase,* decreases in antioxidant protein, *peroxiredoxin-4*, as well as decreases in mitochondrial proteins, including *aldehyde dehydrogenase-2*, o*rnithine aminotransferase*, and *succinate dehydrogenase ubiquinone flavoprotein subunit*. All of these effects were reversed by EGCG pre-treatment. In addition, EGCG attenuated the H_2_O_2_-induced increases of *Type II inositol 3, 4-bisphosphate 4-phosphatase* and relieved its subsequent inhibition of the downstream signalling for Akt and glycogen synthase kinase-3β (GSK-3β)/cyclin D1 in H9c2 cells. Pre-treatment with EGCG or GSK-3β inhibitor (SB 216763) significantly improved the H_2_O_2_-induced suppression on cell viability, phosphorylation of pAkt (S473) and pGSK-3β (S9), and level of cyclin D1 in cells.

**Conclusions:**

Collectively, these findings suggest that EGCG blunts the H_2_O_2_-induced oxidative effect on the Akt activity through the modulation of PIP3 synthesis leading to the subsequent inactivation of GSK-3β mediated cardiac cell injury.

## Background

Oxidative stress has been associated with hypoxia or myocardial ischemia, and likely contributes to the progression of cardiovascular diseases [1]. Accumulating evidence also indicates that redox-sensitive signalling pathways via the effects of generation of reactive oxygen species (ROS) or reactive nitrogen species (RNS) or reactive lipid derived aldehydes (LDAs) are essentially involved in the pathological stress of heart cells [2]. Accordingly, molecular targeting for anti-oxidative interventions on redox signalling pathways may provide a therapeutic approach to ameliorate the risk and progression for heart diseases.

Myocardial ischemia injury involving brief regional ischemia followed by prolonged reperfusion (IR) is the result of an imbalance between myocardial oxygen supply and demand [3]. Such myocardial ischemia stress can cause oxidative stress in myocardium, in which the diminished cellular antioxidant defence system accompanied by the increased ROS production triggers the irreversible cell death [4-6]. However, the detailed mechanism of ROS-induced cardiac cell death during myocardial IR injury remains to be determined. A cell line of H9c2 rat cardiomyoblasts treated with H_2_O_2_ has been used as an in vitro cellular model for cardiac tissues in response to oxidative stress associated with heart IR injury [7-11]. Using this H_2_O_2_-induced oxidative stress model, several studies using proteomics analyses have been reported to identify target proteins associated with oxidative stress with or without antioxidant intervention [7-9].

Green tea polyphenols (GTPs), including epicatechin (EC), epigallocatechin (EGC), epicatechin-3-gallate (ECG), and epigallocatechin-3-gallate (EGCG), have potent properties of antioxidant and radical-scavenger, which may partially account for their anti-atherogenic effects [12-14]. EGCG is the most physiologically potent compound, and predominantly accounts for the biological effects of green tea [15]. Although studies have provided convincing evidence to support the cardioprotective effects of GTPs, the end effectors that mediate cardiac protection are only beginning to be addressed.

The present study aimed to seek a deeper elucidation of the potential proteins for the EGCG-mediated cardioprotection against the H_2_O_2_-induced oxidative stress in H9c2 rat cardiomyoblasts by using a proteomics study. Differential protein expression in control cells with or without treatment were distinguished by two-dimensional electrophoresis (2-DE). After image analysis, the proteins were co-detected, normalized, and quantified. Protein spots cutting off with 1.5 fold difference were selected for protein identification with matrix-assisted laser desorption ionization-time of flight mass spectrometry (MALDI-TOF MS) by peptide mass fingerprinting. The proteins identified were then used to generate an interaction map and to establish interaction networks. Based on the hypothetical model with interaction networks, the present study proposed a putative mechanism for EGCG-induced antioxidant intervention on the H_2_O_2_-induced oxidative stress in H9c2 cells.

## Methods

### Chemicals and reagents

H9c2 cell lines were purchased from American Type Culture Collection (ATCC, CRL-1446) (Rockville, MD). All reagents used were ACS or MB grade. EGCG, purchased from Sigma, was prepared as a stock solution of 10 mM by dissolving the compound in deionized water.

### Cell culture, EGCG and/or H_2_O_2_ treatments, MTT assay

H9c2 cells were cultured in Dulbecco’s modified essential medium (DMEM, Gibco, Invitrogen Taiwan Ltd., Taipei, Taiwan) containing 10% fetal bovine serum (FBS) (Gibco), 25 mM D-glucose, 2 mM L-glutamine, 1 mM sodium pyruvate, 1% streptomycin (100 μg/ml) (Gibco), and 1% penicillin (100 U/ml) (Gibco) at pH 7.4 in a 5% CO_2_ incubator at 37°C. Cell viability was measured using the MTT (3-(4, 5-dimethylthiazol-2-yl)-2,5-diphenyltetrazolium bromide) cell proliferation assay (ATCC, Manassas, VA, USA). Cells (10^5^) were seeded onto 6-cm plates in DMEM-10% FBS. After adhering overnight, the cells were changed to serum-free medium with or without EGCG for 30 min in a 5% CO_2_ incubator at 37°C and then washed with phosphate buffer solution (PBS). The washed cells were treated with different concentrations of H_2_O_2_ in serum-free DMEM for 30 min in a 5% CO_2_ incubator at 37°C. After washing with PBS, the cells were incubated in serum-free DMEM for 24 h in a 5% CO_2_ incubator at 37°C. After 24 h incubation, MTT was then added to the cells at a final concentration of 0.5 mg/ml and the mixture was incubated at 37°C for 4 hours. The optical density of the purple MTT formazan product was measured at 570 nm using a microplate reader.

### Determination of cellular Ca^2+^ levels

Fura 2-AM (fura 2-tetra-acetoxymethyl ester; Molecular Probes, Eugene, OR) was used as the fluorescent indicator. H9c2 cells were dissolved in PBS containing 2 mM fura 2-AM and incubated for 45 min at room temperature and then for 30 min at 37°C, during which time the fura 2-AM was trapped inside by esterase cleavage. The cells were then washed twice with PBS and diluted to a density of 2×10^6^ cells/ml in PBS. Recordings were made in a Perkin-Elmer LS 50B spectrofluorimeter equipped with an accessory to measure Ca^2+^ (Beaconsfield, Buckinghamshire, England). The dye trapped inside the cells was excited every second by exposure to alternating 340 and 380 nm light beams and the intensity of light emission at 510 nm was measured, allowing the monitoring of both the light intensity and the 340 nm fluorescence/380 nm ratio (F340/F380). EGCG was added to the cuvette using a minimum 100-fold concentrated stock solution to avoid large volume variations [16]. The 340/380 ratio (R) was calculated and converted to the corresponding levels of [Ca^2+^]_i_ as described by Grynkiewicz et al. [17], using a Kd of 0.14 μM:

$${\left[{\mathrm{Ca}}^{2+}\right]}_{i}=\mathrm{Kd}*\left(R‒\mathrm{Rmin}\right)/\left(\mathrm{Rmax}‒R\right)*{\mathrm{Sf}}_{2}/{\mathrm{Sb}}_{2}$$

where Rmin and Rmax are the ratios measured by the release of intracellular dye with 2 mM EGTA in 0.1% Triton X-100 (R_min_) followed by the addition of 2.1 mM Ca^2+^ (R_max_), whereas Sf_2_/Sb_2_ is the ratio of the 380 nm signals in Ca^2+^-free and Ca^2+^-replete solutions, respectively.

### Measurement of intracellular ROS generation by fluorescence spectrophotometry

Intracellular ROS levels were assessed using 2’, 7’-dichlorofluorescein diacetate (DCF-DA) [18]. Cells (1.2 × 10^6^) loaded with DCF-DA in 3 ml PBS at a final concentration of 10 μM were incubated at 37°C for 1 h. After incubation, the cells were then washed three times with PBS by centrifugation at 300 × g at 4°C for 5 min. The cells re-suspended with PBS and brought to a density of 10^5^ cells/ml were measured for DCF-DA fluorescence changes every 10 min after the addition of H_2_O_2_ or EGCG by fluorescence spectrophotometry. The fluorescence excitation maximum for DCF-DA was 495 nm, and the corresponding emission maximum was 527 nm.

### Sample preparation and two-dimensional electrophoresis

After treatment, the cells were washed twice with cold PBS and lysed in 2-DE lysis buffer containing 7 M urea, 2 M thiourea, 4% (w/v) 3-[(3-cholamidopropyl) dimethylammonio]-1-propane sulfonate, 2% immobilized pH gradient (IPG) buffer (GE Healthcare UK Ltd., England) and 40 mM DTT. Protein concentration was determined by Bradford protein assay kit (Bio-Rad) according to manufacturer’s procedure. Immobilized nonlinear pH gradient strips (pH 4–7, 24 cm) were rehydrated with 450 μg protein at room temperature overnight (at least 12 h). Isoelectric focusing was then performed using an Ettan IPGphor 3 instrument (GE Healthcare) for a total of 60 kVh at 20°C. After isoelectric focusing, strips were equilibrated in 6 M urea, 75 mM Tris–HCl (pH 8.8), 29.3% (v/v) glycerol, 2% (w/v) SDS and 0.002% (w/v) bromophenol blue with 1% (w/v) DTT for 15 min and then in the same buffer containing 2.5% (w/v) iodoacetamide for 15 min. The equilibrated IPG strips were transferred onto 10% polyacrylamide gels and sealed with 0.5% (w/v) low-melting-point agarose in SDS running buffer containing 0.02% (w/v) bromophenol blue. The gels were run in an Ettan DALTsix electrophoresis system (GE Healthcare) at 40 mA per gel at 20°C until the dye reached the bottom of the gels.

### Gels staining, image analysis and MALDI-TOF MS analysis

After the electrophoresis, gels were stained with Bio-safe™ Coomassie G-250 Stain (Bio-Rad) according to the manufacturer’s protocol. Then, stained gels were scanned with Scanmaker 9800XL (Microtek) using a resolution of 300 dpi. Spot detection, gel matching, and spot quantification were performed by using ImageMaster™ 2D Platinum 7.0 (GE healthcare). The criteria used for selection of candidate protein spots were: (1) the protein spots with >1.5-fold increased or decreased intensity between H_2_O_2_ group and control group (Ctrl.), and (2) the protein spots with >1.5-fold recovery between EGCG + H_2_O_2_ group and H_2_O_2_ group. The proteins of interest were then excised, destained, dehydrated and in-gel digested with trypsin. The resulting peptides were concentrated using Zip-Tip C18 (Millipore). 1 μ1 of peptide was mixed with 1 μ1 of matrix solution (α-cyano-4-hydroxycinnamic acid, 5 mg/mL in 50% (v/v) acetonitrile/0.1% (v/v) trifluoroacetic acid), spotted onto a target plate and subjected to mass spectrometer. The mass fingerprint search was using the MASCOT search engine (Matrix Science, London, UK; http://www.matrixscience.com) against SwissPort/TrEMBL database. The parameters used for searching were: *Rattus*; allow one missed cleavage by trypsin; carbamidomethylation of cysteine, partial methionine oxidation and mass tolerance of 100 ppm. Proteins identification was based on MASCOT Mowse scores (p < 0.05) and the calculated MW and pI had to be in common with the observed MW and pI on 2-DE.

### Gels staining, image analysis and MALDI-TOF MS analysis

After the electrophoresis, gels were stained with Bio-safe™ Coomassie G-250 Stain (Bio-Rad) according to the manufacturer’s protocol. Then, stained gels were scanned with Scanmaker 9800XL (Microtek) using a resolution of 300 dpi. Spot detection, gel matching, and spot quantification were performed by using ImageMaster™ 2D Platinum 7.0 (GE healthcare). The criteria used for selection of candidate protein spots were: (1) the protein spots with >1.5-fold increased or decreased intensity between H_2_O_2_ group and control group (Ctrl.), and (2) the protein spots with >1.5-fold recovery between EGCG + H_2_O_2_ group and H_2_O_2_ group. The proteins of interest were then excised, destained, dehydrated and in-gel digested with trypsin. The resulting peptides were concentrated using Zip-Tip C18 (Millipore). 1 μ1 of peptide was mixed with 1 μ1 of matrix solution (α-cyano-4-hydroxycinnamic acid, 5 mg/mL in 50% (v/v) acetonitrile/0.1% (v/v) trifluoroacetic acid), spotted onto a target plate and subjected to mass spectrometer. The mass fingerprint search was using the MASCOT search engine (Matrix Science, London, UK; http://www.matrixscience.com) against SwissPort/TrEMBL database. The parameters used for searching were: *Rattus*; allow one missed cleavage by trypsin; carbamidomethylation of cysteine, partial methionine oxidation and mass tolerance of 100 ppm. Proteins identification was based on MASCOT Mowse scores (p < 0.05) and the calculated MW and pI had to be in common with the observed MW and pI on 2-DE.

### Real-time polymerase chain reaction

Total RNA was isolated using TRIzol® Reagent (Invitrogen) according to the manufacturer’s protocol. For reverse transcription, 2 μg of total RNA was used for reverse transcription with Moloney Murine Leukemia Virus Reverse Transcriptase (M-MLV-RT) (Genemark) using oligo-dT. Samples were run in triplicate using the SYBR qPCR Kit (Genemark) and the ABI Prism 7300 Sequence Detection System and software (Applied Biosystems). The primers used for qPCR were listed in Table 1.

**Table 1:** Primers used for real time quantitative PCR to detect gene expression in H9c2 cells

**Accession no.**	**mRNA name**	**Primer sequence (5’ → 3’)**	**Size of products (bp)**
NM_032416.1	Aldehyde dehydrogenase 2 family (mitochondrial) (Aldh2)	Forward: TGGCTGATCTCATCGAACGG (360–379)	134
		Reverse: CCAGCCAGCATAATAGCGGA (493–474)	
NM_012554.3	Enolase 1, (alpha) (Eno1)	Forward: CCTACTGCCAGAACTTCACCA (102–122)	208
		Reverse: GAGACACCCTTCCCCATGAA (309–290)	
NM_057141.1	Heterogeneous nuclear ribonucleoprotein K (Hnrnpk)	Forward: CACCTTGCTTTGTGGTCACTG (1700–1720)	232
		Reverse: TTAGTTTAGGGGTGGGCTGG (1931–1912)	
NM_001007149.1	Staufen, RNA binding protein, homolog 2 (Drosophila) (Stau2)	Forward: CAGAGCGGGGTCATTTCTCG (25–44)	220
		Reverse: GGATGCTATGGAAACGGGCT (244–225)	
NM_022521.3	Ornithine aminotransferase (Oat)	Forward: CAGGGTGAAGCGGGTGTTAT (803–822)	262
		Reverse: CGTGCTCGCCTGGTTTAATG (1064–1045)	
NM_053917.1	Inositol polyphosphate-4-phosphatase, type II (Inpp4b)	Forward: ATGGAAAAGATGCCGCCTGA (2739–2758)	239
		Reverse: TCGTCTCTCAGGATGGAGCA (2977–2958)	
NM_053512.2	Peroxiredoxin 4 (Prdx4)	Forward: GCCAAGATTTCCAAGCCAGC (268–287)	284
		Reverse: CTTATTGGCCCCAGTCCTCC (551–532)	
NM_130428.1	Succinate dehydrogenase complex, subunit A, flavoprotein (Fp) (Sdha)	Forward: ATGGGCGAACCTACTTCAGC (793–812)	84
		Reverse: AAGGTAAACCAGCCCGAGTG (876–857)	

### Western blot analysis

After treatment, the cells were washed twice with cold PBS and lysed in cell lysis buffer containing 20 mM Tris–HCl (pH 7.5), 150 mM NaCl, 1% Triton X-100, 1 mM phenylmethanesulfonyl fluoride, 2 mM 4-(2-aminoethyl) benzenesulfonyl fluoride hydrochloride, 0.3 μM aprotinin, 130 μM bestatin, 14 μM proteinase inhibitor E-64, 1 mM EDTA, 1 μM leupetin and 1% phosphatase inhibitor cocktail 2/3 (Sigma). Protein concentration was determined by Bradford protein assay kit (Bio-Rad). One hundred microgram of samples were resolved on 12% SDS-PAGE gels and then transferred onto a PVDF membrane. The immuo-blotting procedure was as described previously [11]. The membranes were blocked with 5% bovine serum albumin (BSA) and incubated with anti-Inpp4b (Santa Cruz; 1: 1000 dilution), anti-phospho-AKT (Ser473) (Sigma, 1: 500 dilution), anti-phospho-AKT (Thr308) (Santa Cruz, 1: 500 dilution), anti-phospho-GSK-3β (Ser9) (Santa Cruz; 1: 1000 dilution), anti-cyclin D1 (Santa Cruz; 1: 1000 dilution) and anti-GAPDH antibody (Santa Cruz; 1: 1000 dilution), followed by incubation with AP-conjugated anti-rabbit or anti-mouse IgG secondary antibodies (Santa Cruz; 1:5000). Proteins specifically recognized by the antibody were visualized using the 5-bromo-4-chloro-3-indolyl phosphate-nitro blue tetrazolium substrate kit (invitrogen). Band intensities were quantified using Quantity One software (Bio-Rad).

### Measurements of Aldh activity

*Aldh* activity was measured at 25°C in 33 mM sodium pyrophosphate containing 0.8 mM NAD^+^, 15 μM propionaldehyde and 0.1 ml of cellular extract (50 μg soluble protein). Propionaldehyde, the substrate of *Aldh*, was oxidized into propionic acid by *Aldh*, while NAD^+^ was reduced to NADH to quantitatively indicate the *Aldh* activity. Production of NADH was determined by spectrophotometric absorbance at 340 nm. *Aldh* activity was expressed as nmol NADH/min per mg protein. An extinction coefficient of 6.22/mM per cm for NADH was used for the calculation of reaction rates [19].

#### Statistical analysis

Excel 2013 (Microsoft office) was used to perform statistical analyses. Quantitative values are presented as mean ± standard error (mean ± SEM). Statistical significance between more than two groups was tested using one-way ANOVA, while comparisons between two groups were performed using Student’s t test. Differences were considered to be statistically significant when p < 0.05 or less.

## Results

### The proteomic strategy used to evaluate EGCG-mediated cardioprotection against H_2_O_2_-induced oxidative stress in H9c2 rat cardiomyoblasts

**Figure 1:**
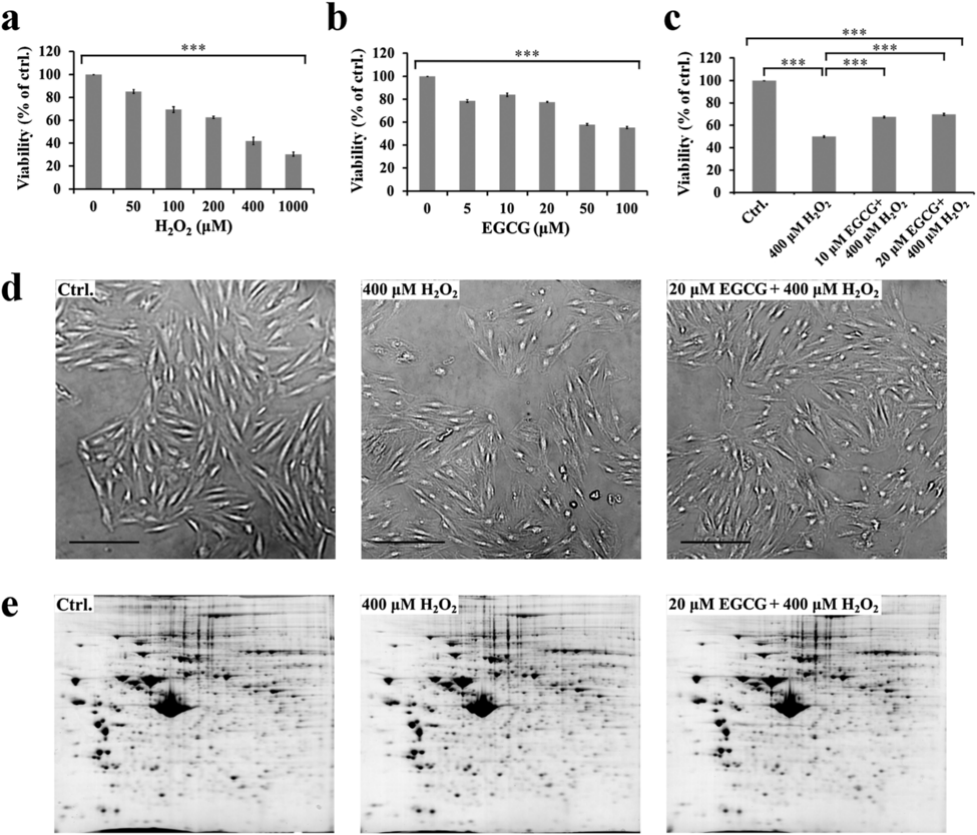
**A proteomic study of the effects of EGCG on H**_**2**_**O**_**2**_**-induced oxidative stress in H9c2 cells. (a)** MTT assay of cell viability after incubation with 0, 50, 100, 200, 400 and 1000 μM H_2_O_2_ for 30 min **(b)** MTT assay of cell viability after incubation with 0, 5, 10, 20, 50 and 100 μM EGCG for 30 min **(c)** H9c2 cells were pre-treated with 10 and 20 μM EGCG for 30 min followed by 400 μM H_2_O_2_ for 30 min, cell viability was measured by MTT assay **(d)** Phase contrast microscopy showing cell morphology of H9c2 cells in the condition of control (left), 400 μM H_2_O_2_ exposure for 30 min (middle), and 20 μM EGCG pre-treatment for 30 min followed by 400 μM H_2_O_2_ exposure for 30 min (right). (Scale Bar = 200 μm). **(e)** 2-DE gel images showing protein distribution in the condition of control (left), 400 μM H_2_O_2_ exposure for 30 min (middle), and 20 μM EGCG pre-treatment for 30 min followed by 400 μM H_2_O_2_ exposure for 30 min (right). In **a**, the values are the mean ± SEM, with ***p < 0.001.

In this study, H_2_O_2_ treatment of H9c2 rat cardiomyocytes was used as a model for oxidative stress associated with heart IR injury. Upon H_2_O_2_ treatment from 0 to 1000 μM for 30 min, a dose-dependent decrease in cell viability occurred in H9c2 cells with a 50% decrease of cell viability occurred at 400 μM H_2_O_2_ (Figure 1a). On the other hand, the toxicity of EGCG yielding 50% cell death for H9c2 cells was found to appear at 50 μM (Figure 1b). EGCG pre-treatment with 10 or 20 μM for 30 min effectively improved viability of cells in prior to their exposure to 400 μM H_2_O_2_ (Figure 1c). To understand further the molecular events for EGCG-mediated anti-oxidative intervention on the H_2_O_2_-induced oxidative stress, H9c2 cells cultured in the medium of control, 400 μM H_2_O_2_ with or without 20 μM EGCG pre-treatment (Figure 1d) were used to differentiate their protein expression profile by 2-DE analyses (Figure 1e).

### 2-DE analysis on differential protein expression in control, and H_2_O_2_-treated H9c2 cells with and without EGCG pretreatment

**Figure 2:**
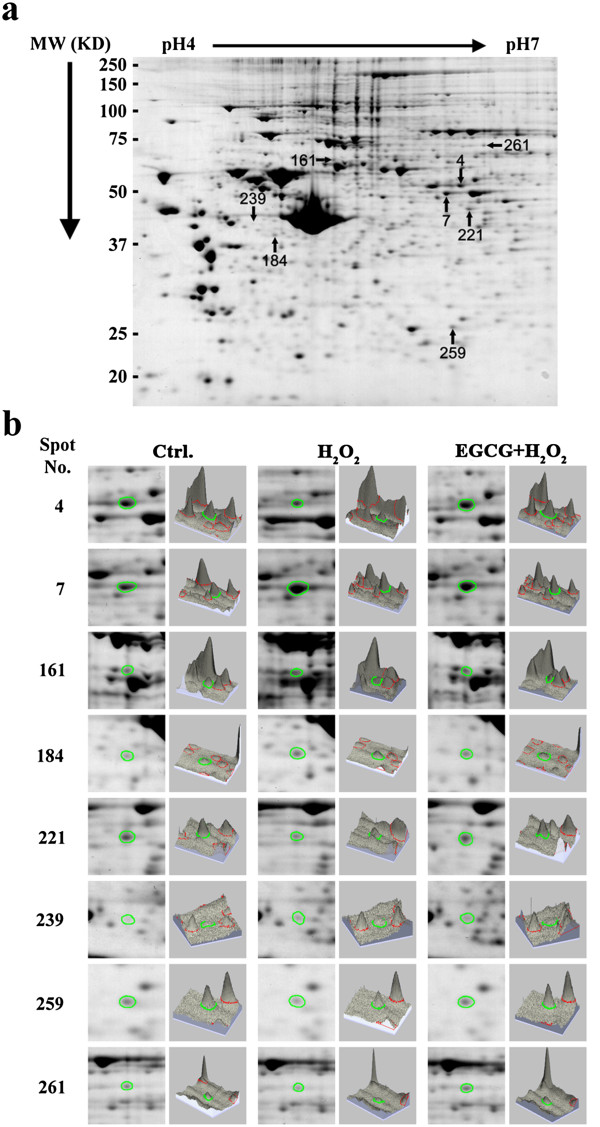
**2D analysis. (a)** A coomassie blue stained of 2-DE map showing the differentially protein spot profiles in H9c2 cells. The differentially expressed proteins are annotated with spot numbers. **(b)** Representative images of each spot in 2-DE map (left) and three-dimensional spot images (right).

**Figure 3:**
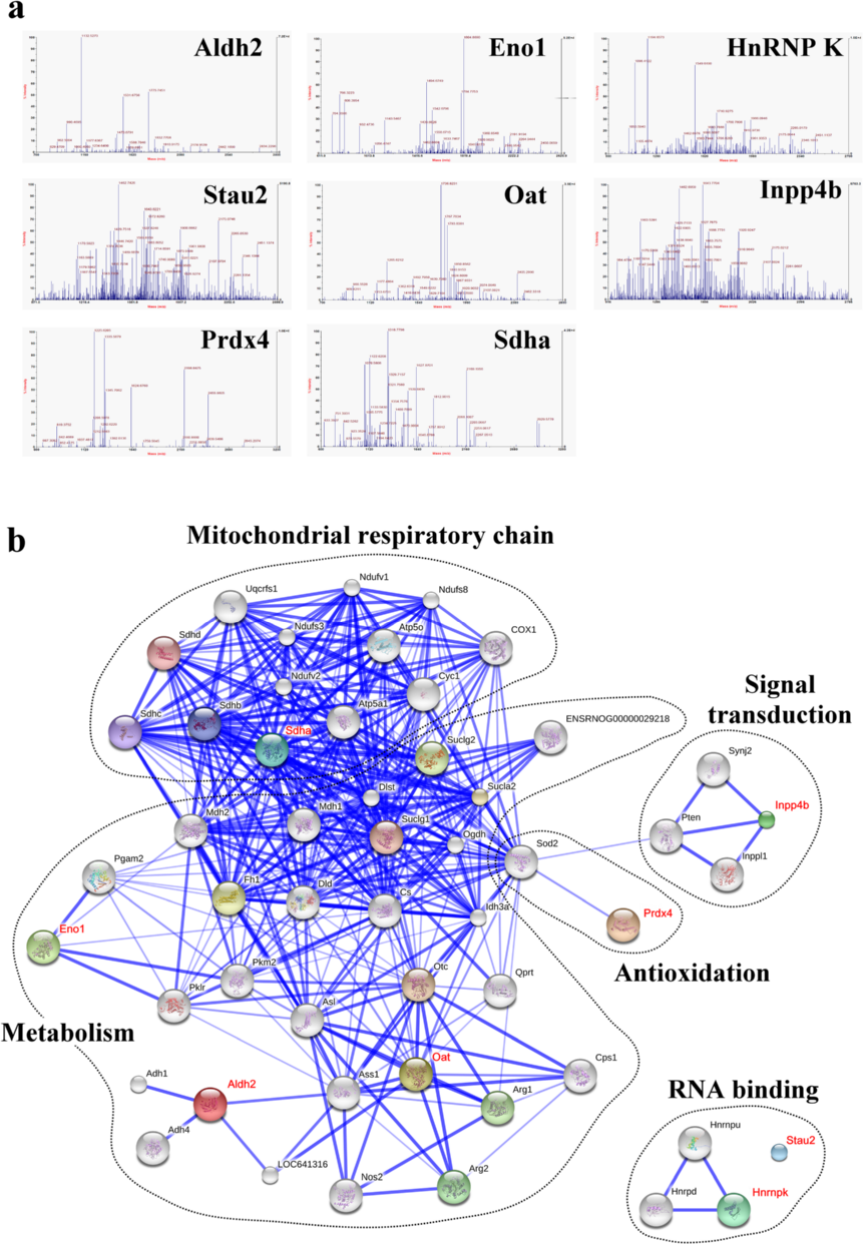
**Molecular identification and hypothetical protein-protein interactions. (a)** Peptide mass fingerprinting of Aldh2, Eno1, HnRNP K, Stau2, Oat, Inpp4b, Prdx4 and Sdha were identified by MALDI-TOF. **(b)** The identified proteins in Table 2 were input into STRING database (http://string-db.org/) and an interaction map was generated.

2-DE global protein expression analysis resolved more than 330 protein spots showing differential expression among three conditions (Figure 2a). In order to understand H9c2 cells in response to H_2_O_2_-induced oxidative stress and EGCG-mediated antioxidant interventions, the criteria setting with >1.5-fold increased or decreased intensity between H_2_O_2_ group and control group, and >1.5-fold recovery between EGCG pretreatment group and H_2_O_2_ group were used to select candidate protein spots on the 2-DE gels. According to the thresholded settings, 8 protein spots confirmed by three-dimensional image analysis (Figure 2b) were selected for protein identification with MALDI-TOF mass spectrometry by peptide mass fingerprinting (Figure 3a). Identified proteins were listed in Table 2. To establish a hypothetical model for interaction networks, the proteins identified were imported into the EMBL Search Tool for the Retrieval of Interacting Proteins (STRING) database (http://string-db.org/) to generate an interaction map (Figure 3b). According to functionally annotations derived from the reported database, these differentially expressed proteins are implicated in cellular energetic metabolism, including: *α-enolase (Eno1)*, *aldehyde dehydrogenase-2 (Aldh2)*, and o*rnithine aminotransferase (Oat)*, mitochondrial electron transfer, i.e. *succinate dehydrogenase ubiquinone flavoprotein subunit (Sdha)*, redox regulation, i.e. *peroxiredoxin-4 (Prdx4)*, Akt signal transduction, i.e. *Type II inositol 3,4-bisphosphate 4-phosphatase (Inpp4b)*, RNA binding, i.e. *heterogeneous nuclear ribonucleoprotein K (HnRNP K)* and *Staufen homolog 2 (Stau2)* (Table 2).

**Table 2:** List of identified differentially expressed proteins obtained with 2D-DIGE coupled with MALDI-TOF MS analysis

**Spot no.**	**Swiss-Prot no.**	**Protein name**	**MW**	**p** ***I***	**No. match peptides**	**Cov.(%)**	**Score**	**Function**	**H**_**2**_**O**_**2**_**/Ctrl**	**EGCG/H**_**2**_**O**_**2**_
***Constitutively upregulated***										
7	P04764	Alpha-enolase (Eno1)	47,440	6.16	18/48	43	153/51	Metabolism	+1.88	−1.77
184	Q68SB1	Double-stranded RNA-binding protein Staufen homolog 2 (Stau2)	62,870	9.54	14/61	23	59/51	RNA binding	+1.75	−2.46
239	Q9QWG5	Type II inositol 3,4-bisphosphate 4-phosphatase (Inpp4b)	106,205	5.86	12/44	14	57/51	Signal transduction	+2.24	−1.80
***Constitutively downregulated***										
4	P11884	Aldehyde dehydrogenase, mitochondrial (Aldh2)	56,966	6.63	15/40	33	141/51	Metabolism	−1.99	+2.02
161	P61980	Heterogeneous nuclear ribonucleoprotein K (Hnrnpk)	51,230	5.39	10/39	28	76/51	RNA binding	−2.43	+2.01
221	P04182	Ornithine aminotransferase, mitochondrial (Oat)	48,701	6.53	13/38	30	123/51	Metabolism	−1.94	+1.87
259	Q9Z0V5	Peroxiredoxin-4 (Prdx4)	31,216	6.18	17/34	57	221/51	Antioxidation	−3.71	+2.89
261	Q920L2	Succinate dehydrogenase [ubiquinone] flavoprotein subunit, mitochondrial (Sdha)	72,596	6.75	31/52	51	305/51	Electron transport	−3.21	+2.34

Average ratios of differential expression (p < 0.05) across H9c2 cells, H9c2 cells treated with and/or without 10 to 20 μM EGCG pre-treatment followed by 400 μM H_2_O_2_ exposure were calculated from triplicate gels.

### Effects of H_2_O_2_ and EGCG on oxidative stress associated with cellular metabolism

**Figure 4:**
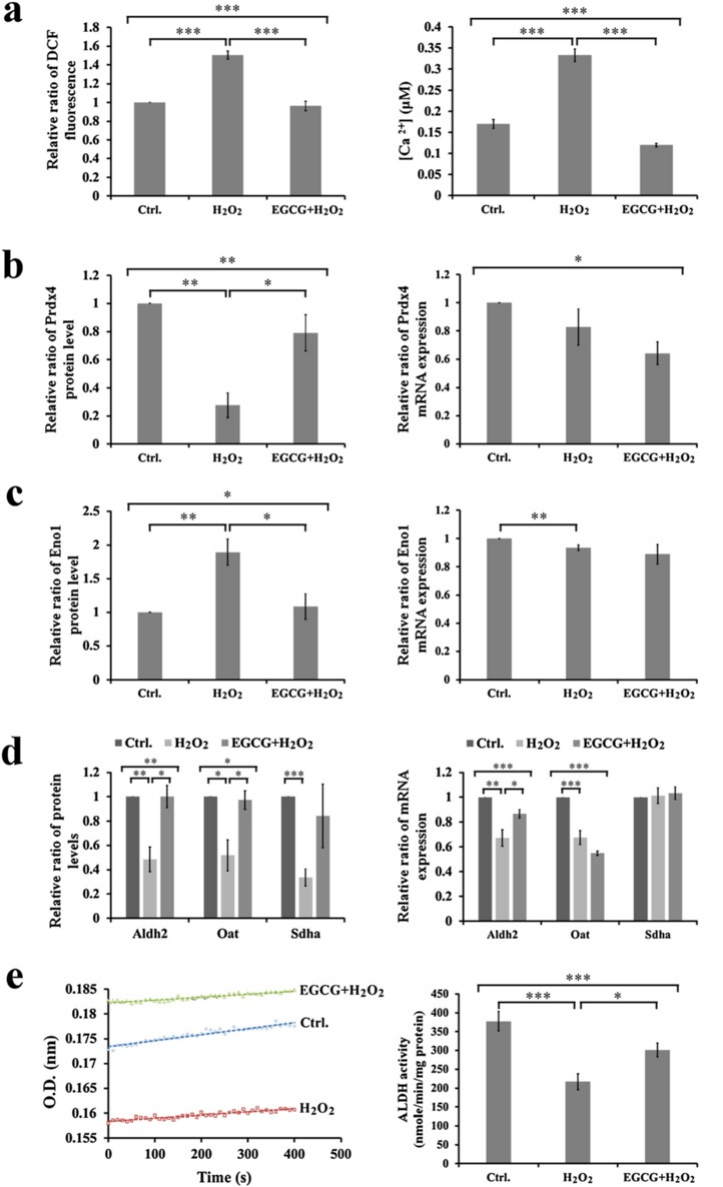
**Effects of H**_**2**_**O**_**2 **_**and EGCG on oxidative stress associated with cellular metabolism. (a)** Measurements of intracellular ROS formation by DCF-DA in H9c2 cells (left). Cellular Ca^2+^ levels were measured using the Fura-2 fluorescence dye (right). **(b)** Representative result of Prdx4 protein levels in 2-DE gel and mRNA expression **(c)** Representative result of Eno1 protein levels in 2-DE gel and mRNA expression **(d)** Representative results of Aldh2, Oat and Sdha protein levels in 2-DE gel and mRNA expression. **(e)** ALDH activity was measured by spectrophotometer at 340 nm (left). Quantitative results showed protective effect of EGCG in H_2_O_2_-induced H9c2 cells (right). All values are the mean ± SEM, with *p < 0.05; **p < 0.01; ***p < 0.001.

H_2_O_2_ exposure increased oxidative stress in H9c2 cells, evidenced by increases in ROS and cytosolic Ca^2+^ overload (Figure 4a). In addition, H_2_O_2_ exposure decreased the protein level but not mRNA expression for antioxidant protein, i.e. *Prdx4*, in H9c2 cells; whereas, EGCG pretreatment prevented the decrease of the protein level without effect on the mRNA expression for *Prdx4* in H_2_O_2_-treated cells (Figure 4b). When H9c2 cells were treated with H_2_O_2_, the protein level of glycolytic protein, *Eno1*, was increased with its decreased mRNA expression in cells (Figure 4b). EGCG pretreatment reversed the H_2_O_2_-decreased protein level with no effect on the mRNA expression for *Eno1* in the H_2_O_2_-treated cells (Figure 4c). In contrast, mitochondrial proteins involved in aerobic energy production, including *Aldh2*, *Oat*, and *Sdha* were decreased in H9c2 cells with the H_2_O_2_-induced oxidative stress, but not changed in the H_2_O_2_-treated cells with EGCG pretreatment, as compared to cells in the control condition (Figure 4d). For these mitochondrial proteins, only *Aldh2* mRNA expression was decreased by H_2_O_2_-induced oxidative stress but recovered by EGCG pretreatment (Figure 4d). In addition, total cellular *Aldh* activity (nmole/min/mg protein) was measured as 377.6 ± 26.0, 217.0 ± 21.1, and 301.5 ± 18.7 in H9c2 cells under the conditions of control, H_2_O_2_ treatment with and without EGCG, respectively (Figure 4e). These results suggested that under the H_2_O_2_-induced oxidative stress, H9c2 cells undergo the inflicted cellular changes of energy production by switching aerobic metabolism to anaerobic metabolism. Moreover, EGCG pretreatment could induce antioxidant intervention and protect cardiac cells from the H_2_O_2_-induced oxidative stress.

### Effects of H_2_O_2_ and EGCG on PI3K/Akt/GSK3β signaling pathway

**Figure 5:**
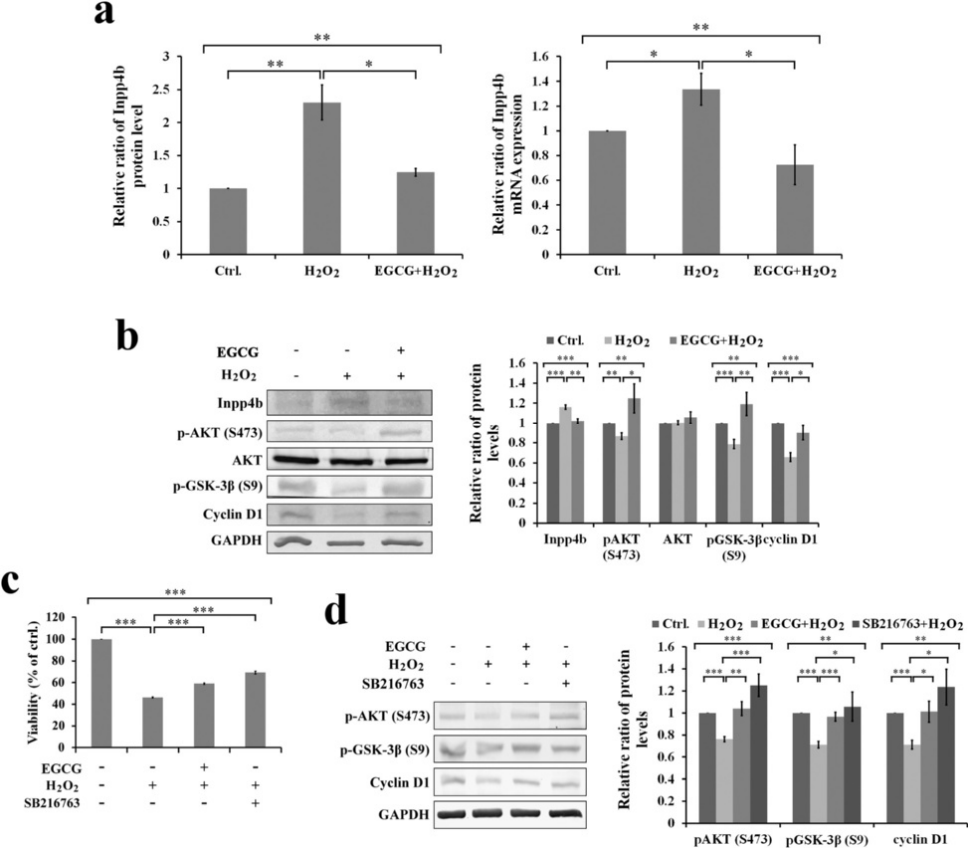
**Effects of H**_**2**_**O**_**2 **_**and EGCG on PI3K/AKT/GSK3β signaling pathway. (a)** Representative result of Inpp4b protein level in 2-DE gel and mRNA expression **(b)** Western blotting (right) and quantitative analysis (left) showing effects of EGCG on Inpp4b, p-AKT (S473), AKT, p-GSK-3β (S9), cyclin D1 in H_2_O_2_-induced H9c2 cells. **(c)** MTT assay showing the improvement of H_2_O_2_-suppressed cell viability by EGCG and/or GSK-3β inhibitor, SB216763 pre-treatment in H_2_O_2_-induced H9c2 cells. **(d)** Western blotting (right) and quantitative analysis (left) showing effects of EGCG and/or GSK-3β inhibition by GSK-3β inhibitor, SB216763, on the phosphorylation of Akt (S473), GSK-3β (S9) and cyclin D1 in H_2_O_2_-induced H9c2 cells. All values are the mean ± SEM, with *p < 0.05; **p < 0.01; ***p < 0.001.

*Inpp4b*, one of the enzymes involved in membrane phosphatidylinositol (PI) metabolism, has been shown to act as one of phosphoinositide 3-kinase (PI3K) inhibitors for the subsequent activation of Akt prosurvival signalling pathway [20-22]. In the present study, *Inpp4b* protein level and mRNA expression were increased when cells were exposed to H_2_O_2_ (Figure 5a). Concomitantly, the levels of phosphorylated Akt (S473), phosphorylated GSK-3b (S9), and cyclin D1 were decreased in H_2_O_2_-treated cells (Figure 5b). This result is consistent with the finding that oxidative stress regulates the activity of the cell survival factor Akt through the regulation of PI(3,4,5)P3 and PI(3,4)P2 synthesis [23]. In addition, EGCG pretreatment counteracted the H_2_O_2_-increased *Inpp4b* expression in H9c2 cells (Figure 5a).

Recently, we have shown that the Akt prosurvival pathway associated with glycogen synthase kinase-3β (GSK-3β) signalling takes part in EGCG-mediated cardoioprotection in an H_2_O_2_-induced H9c2 cell injury [11]. Consistently, immunoblot analyses showed that EGCG attenuated the H_2_O_2_-induced increases *Inpp4b* and relieved its subsequent inhibition of the downstream signalling for Akt and GSK-3β/cyclin D1 in H9c2 cells (Figure 5b). Pre-treatment with EGCG or GSK-3β inhibitor (SB 216763) significantly improved the H_2_O_2_-induced suppression on cell viability (Figure 5c), phosphorylation of pAkt (S473) and pGSK-3β (S9), and level of cyclin D1 (Figure 5d) in cells.

**Figure 6:**
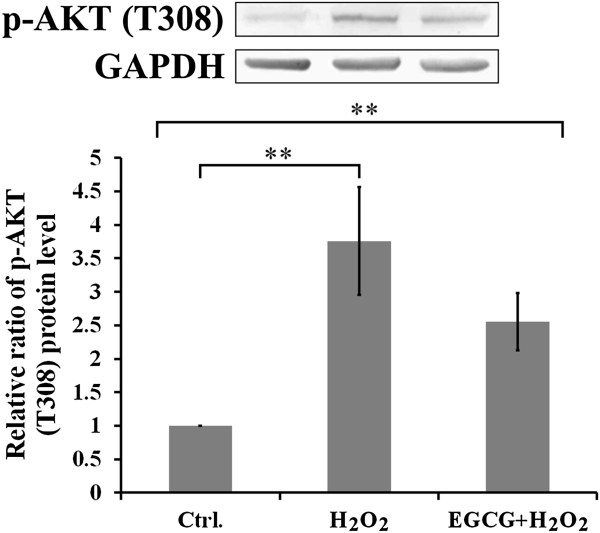
**Effects of H**_**2**_**O**_**2 **_**and EGCG on p-AKT (T308).** Western blotting (upper) and quantitative analysis (lower) showing effects of EGCG on the p-AKT (T308) in H_2_O_2_-induced H9c2 cells. All values are the mean ± SEM, with **p < 0.01.

Figure 6 shows effects of H_2_O_2_ and EGCG on the Thr308 (T308) phosphorylation of Akt in H9c2 cells. In contrast to Akt phosphorylation at S473, H_2_O_2_ exposure significantly increased Akt phosphorylation at T308 in H9c2 cells. With EGCG pretreatment, T308 phosphorylation was suppressed by 20% in H_2_O_2_-treated H9c2 cells.

## Discussion

Previously, we have demonstrated the cardio-protection of green tea polyphenols (GTPs) against oxidative stress associated with myocardial ischemic injury by reducing cytosolic Ca^2+^ overload and generation of ROS via the Akt/GSK-3β/β-catenine and caveolae signaling in a rat surgical model of myocardial ischemia and in an H_2_O_2_-induced oxidative stress model of H9c2 rat cardiomyoblasts [11,24-26]. In the present study, using the H9c2 cell model of H_2_O_2_-induced oxidative stress for a proteomics study (Figures 1, 2), we identified proteins involved in energy metabolism, mitochondrial electron transfer, redox regulation, signal transduction, and RNA binding that might take part in EGCG-ameliorating H_2_O_2_-induced injury in H9c2 cells (Figure 3, Table 2).

During hypoxia or ischemia, ATP depletion impairs the Ca^2+^ uptake capacity of the sarcoplasmic reticulum (SR), leading to intracellular Ca^2+^ accumulation [27]. The rise in Ca^2+^ leads to mitochondrial Ca^2+^ accumulation, particularly during reperfusion when oxygen is reintroduced. Reintroduction of oxygen causing damage to the electron transport chain results in increased mitochondrial generation of ROS [27]. Both mitochondrial Ca^2+^ overload and increased ROS can result in opening of the mitochondrial permeability transition pore, which further compromises cellular energetics [28]. In this study, not only increasing ROS formation and cytosolic Ca^2+^ overload (Figure 4a), H_2_O_2_ exposure also resulted in decreasing the level of antioxidant, *Prox4* in H9c2 cells (Figure 4b) as well as altering the expression for cellular energy production by decreasing the expression for mitochondrial metabolism (i.e. *Aldh2*, *Oat*, and *Sdha*) (Figure 4c) but increasing the expression for glycolytic metabolism (i.e. *Eno1*) (Figure 4d). Apparently, oxidative stress strongly correlates with mitochondrial dysfunction and likely contributes to the decline in mitochondrial bioenergetics [29]. Moreover, the activity of ALDH was found to decrease significantly in the H_2_O_2_-treated H9c2 cells (Figure 4e). This might suggest that excessive ROS leading to the formation of potentially toxic aldehydes induces inactivation of *Aldh2* such as to impair the cardiac functions [30]. The present study also showed that EGCG pretreatment prevented the decrease of antioxidant, *Prdx4* in H_2_O_2_-treated H9c2 cells (Figure 4b), and avoided the H_2_O_2_-decreased mitochondrial proteins (Figure 4c) with -increased glycolytic protein, *Eno1* (Figure 4d), as well as ameliorated the *Aldh2* activity during the H_2_O_2_-induced oxidative stress (Figure 4e). Consistently, a study with cultured rat cardiomyocytes exposed to different periods of hypoxia (H), followed by reoxygenation (R), demonstrated that GTPs acts to counteract the H/R damage-induced switch to the biosynthesis of highly unsaturated fatty acids [31], pointing out the importance of GTPs in providing good antioxidant defence not only after, but mainly prior to, the onset of H [31].

It has been shown that oxidative stress induced the PI3K/Akt dependent apoptosis in cardiac cells [32,33]. PI3K produces two lipid products that PI(3,4,5)P3 contributes predominantly to Thr308 (T308) phosphorylation and membrane-associated activation of Akt, but PI(3,4)P2 contributes mostly to Ser473 (S473) phosphorylation and cytoplasmic activation of Akt [34]. In this study, the H_2_O_2_-increased Inpp4b in concomitant with the decrease in protein levels of phosphorylated Akt (S473), phosphorylated GSK-3β (S9), and cyclin D1 appeared in H9c2 cells (Figure 5). This result might also suggest that the H_2_O_2_-induced oxidative stress caused to decrease the level of PI(3,4)P2 for turning on activation of cytosolic Akt phosphorylation at S473 such as to relieve the subsequent inhibition on the downstream target of GSK-3β/cyclin D1 in H9c2 cells (Figure 5). It has been shown that GSK-3 inhibition limits myocardial IR injury and stimulates glycogen synthesis, repartitions glucose away from glycolysis, reduces proton production from glucose metabolism, and attenuates intracellular Ca^2+^ overload [35]. Moreover, several studies have shown that H_2_O_2_-induced oxidative stress can trigger T308 phosphorylation for membrane-associated activation of Akt, by the PI3K dependent pathway in lymphocytes [36-38]. The present study also indicated that H_2_O_2_ exposure modulates the PI3K signalling events for Akt phosphorylation at T308 in H9c2 cells, and this Akt phosphorylation at T308 is partly suppressed by EGCG pretreatment (Figure 6).

The present study using cardiac proteomic analysis has identified EGCG-induced cardio-protection against H_2_O_2_-induced oxidative stress through the Akt/GSK-3β pathway in cultured H9c2 cells. However, the limitation of this study was to identify the modified target proteins associated with anti-oxidative effect of EGCG. The future work using redox proteomics might further help identify and quantify EGCG-mediated changes within the proteome both in redox signaling and under oxidative stress conditions.

## Conclusions

In summary, the results obtained with proteomic analyses that EGCG blunts the H_2_O_2_-induced oxidative effect on the Akt activity through the modulation of PI(3,4)P2 and PI (3,4,5)P3 synthesis leading to the subsequent inhibition of GSK-3β mediated cardiac cell injury.

## Competing interests

The authors declare that they have no competing interests.

## Authors’ contributions

CWC carried out all experiments, and drafted the manuscript. HSR participated in the design of the study, and drafted the manuscript. CCH participated in the measurements of *Aldh* activity. HBD participated in the design of the study. LYM conceived of the study, and participated in the design and coordination and helped to draft the manuscript and final MS submission. All authors read and approved the final manuscript.

## References

[B1] Santos CX Anilkumar N Zhang M Brewer AC Shah AM Redox signaling in cardiac myocytes Free Radic Biol Med 2011 50 777 793 10.1016/j.freeradbiomed.2011.01.003 21236334 PMC3049876

[B2] Wall SB Oh JY Diers AR Landar A Oxidative modification of proteins: an emerging mechanism of cell signaling Front Physiol 2012 3 369 23049513 10.3389/fphys.2012.00369 PMC3442266

[B3] Verdouw PD van den Doel MA de Zeeuw S Duncker DJ Animal models in the study of myocardial ischaemia and ischaemic syndromes Cardiovasc Res 1998 39 121 135 10.1016/S0008-6363(98)00069-8 9764194

[B4] Burgoyne JR Mongue-Din H Eaton P Shah AM Redox signaling in cardiac physiology and pathology Circ Res 2012 111 1091 1106 10.1161/CIRCRESAHA.111.255216 23023511

[B5] Ferdinandy P Schulz R Baxter GF Interaction of cardiovascular risk factors with myocardial ischemia/reperfusion injury, preconditioning, and postconditioning Pharmacol Rev 2007 59 418 458 10.1124/pr.107.06002 18048761

[B6] Anversa P Kajstura J Myocyte cell death in the diseased heart Circ Res 1998 82 1231 1233 10.1161/01.RES.82.11.1231 9633922

[B7] Chou HC Chen YW Lee TR Wu FS Chan HT Lyu PC Timms JF Chan HL Proteomics study of oxidative stress and Src kinase inhibition in H9C2 cardiomyocytes: a cell model of heart ischemia-reperfusion injury and treatment Free Radic Biol Med 2010 49 96 108 10.1016/j.freeradbiomed.2010.04.001 20385227

[B8] Law CH Li JM Chou HC Chen YH Chan HL Hyaluronic acid-dependent protection in H9C2 cardiomyocytes: a cell model of heart ischemia-reperfusion injury and treatment Toxicology 2013 303 54 71 23178681 10.1016/j.tox.2012.11.006

[B9] Chen YW Chou HC Lin ST Chen YH Chang YJ Chen L Chan HL Cardioprotective effects of quercetin in cardiomyocyte under ischemia/reperfusion injury Evid Based Complement Alternat Med 2013 2013 364519 23573126 10.1155/2013/364519 PMC3612448

[B10] Chou HC Chan HL 5-Methoxytryptophan-dependent protection of cardiomyocytes from heart ischemia reperfusion injury Arch Biochem Biophys 2014 543 15 22 24384558 10.1016/j.abb.2013.12.014

[B11] Hsieh SR Hsu CS Lu CH Chen WC Chiu CH Liou YM Epigallocatechin-3-gallate-mediated cardioprotection by Akt/GSK-3beta/caveolin signalling in H9c2 rat cardiomyoblasts J Biomed Sci 2013 20 86 10.1186/1423-0127-20-86 24251870 PMC3871020

[B12] Stangl V Dreger H Stangl K Lorenz M Molecular targets of tea polyphenols in the cardiovascular system Cardiovasc Res 2007 73 348 358 10.1016/j.cardiores.2006.08.022 17020753

[B13] Mak JC Potential role of green tea catechins in various disease therapies: progress and promise Clin Exp Pharmacol Physiol 2012 39 265 273 10.1111/j.1440-1681.2012.05673.x 22229384

[B14] Nakagawa T Yokozawa T Direct scavenging of nitric oxide and superoxide by green tea Food Chem Toxicol 2002 40 1745 1750 10.1016/S0278-6915(02)00169-2 12419687

[B15] L’Allemain G Multiple actions of EGCG, the main component of green tea Bull Cancer 1999 86 721 724 10519963

[B16] Hsu YC Liou YM The anti-cancer effects of (−)-epigallocatechin-3-gallate on the signaling pathways associated with membrane receptors in MCF-7 cells J Cell Physiol 2011 226 2721 2730 10.1002/jcp.22623 21792929

[B17] Grynkiewicz G Poenie M Tsien RY A new generation of Ca^2+^ indicators with greatly improved fluorescence properties J Biol Chem 1985 260 3440 3450 3838314

[B18] Maciel EN Vercesi AE Castilho RF Oxidative stress in Ca(2+)-induced membrane permeability transition in brain mitochondria J Neurochem 2001 79 1237 1245 11752064 10.1046/j.1471-4159.2001.00670.x

[B19] Li SY Li Q Shen JJ Dong F Sigmon VK Liu Y Ren J Attenuation of acetaldehyde-induced cell injury by overexpression of aldehyde dehydrogenase-2 (ALDH2) transgene in human cardiac myocytes: role of MAP kinase signaling J Mol Cell Cardiol 2006 40 283 294 10.1016/j.yjmcc.2005.11.006 16403513

[B20] Agoulnik IU Hodgson MC Bowden WA Ittmann MM INPP4B: the new kid on the PI3K block Oncotarget 2011 2 321 328 21487159 10.18632/oncotarget.260 PMC3248162

[B21] Bozulic L Hemmings BA PIKKing on PKB: regulation of PKB activity by phosphorylation Curr Opin Cell Biol 2009 21 256 261 10.1016/j.ceb.2009.02.002 19303758

[B22] Bozulic L Surucu B Hynx D Hemmings BA PKBalpha/Akt1 acts downstream of DNA-PK in the DNA double-strand break response and promotes survival Mol Cell 2008 30 203 213 10.1016/j.molcel.2008.02.024 18439899

[B23] Ma K Cheung SM Marshall AJ Duronio V PI(3,4,5)P3 and PI(3,4)P2 levels correlate with PKB/akt phosphorylation at Thr308 and Ser473, respectively; PI(3,4)P2 levels determine PKB activity Cell Signal 2008 20 684 694 10.1016/j.cellsig.2007.12.004 18249092

[B24] Hsieh SR Tsai DC Chen JY Tsai SW Liou YM Green tea extract protects rats against myocardial infarction associated with left anterior descending coronary artery ligation Pflugers Arch 2009 458 631 642 10.1007/s00424-009-0655-1 19263074

[B25] Liou YM Hsieh SR Wu TJ Chen JY Green tea extract given before regional myocardial ischemia-reperfusion in rats improves myocardial contractility by attenuating calcium overload Pflugers Arch 2010 460 1003 1014 10.1007/s00424-010-0881-6 20922441

[B26] Liou YM Kuo SC Hsieh SR Differential effects of a green tea-derived polyphenol (−)-epigallocatechin-3-gallate on the acidosis-induced decrease in the Ca(2+) sensitivity of cardiac and skeletal muscle Pflugers Arch 2008 456 787 800 10.1007/s00424-008-0456-y 18231806

[B27] Murphy E Steenbergen C Mechanisms underlying acute protection from cardiac ischemia-reperfusion injury Physiol Rev 2008 88 581 609 10.1152/physrev.00024.2007 18391174 PMC3199571

[B28] Peng TI Jou MJ Oxidative stress caused by mitochondrial calcium overload Ann N Y Acad Sci 2010 1201 183 188 10.1111/j.1749-6632.2010.05634.x 20649555

[B29] Judge S Leeuwenburgh C Cardiac mitochondrial bioenergetics, oxidative stress, and aging Am J Physiol Cell Physiol 2007 292 C1983 C1992 10.1152/ajpcell.00285.2006 17344313

[B30] Wang J Wang H Hao P Xue L Wei S Zhang Y Chen Y Inhibition of aldehyde dehydrogenase 2 by oxidative stress is associated with cardiac dysfunction in diabetic rats Mol Med 2011 17 172 179 20957334 10.2119/molmed.2010.00114 PMC3060979

[B31] Bordoni A Angeloni C Leoncini E Danesi F Maranesi M Biagi PL Hrelia S Hypoxia/reoxygenation alters essential fatty acids metabolism in cultured rat cardiomyocytes: protection by antioxidants Nutr Metab Cardiovasc Dis 2005 15 166 173 10.1016/j.numecd.2004.04.003 15955464

[B32] Zhu Y Shi YP Wu D Ji YJ Wang X Chen HL Wu SS Huang DJ Jiang W Salidroside protects against hydrogen peroxide-induced injury in cardiac H9c2 cells via PI3K-Akt dependent pathway DNA Cell Biol 2011 30 809 819 10.1089/dna.2010.1183 21563965

[B33] Angeloni C Spencer JP Leoncini E Biagi PL Hrelia S Role of quercetin and its in vivo metabolites in protecting H9c2 cells against oxidative stress Biochimie 2007 89 73 82 10.1016/j.biochi.2006.09.006 17045724

[B34] Scheid MP Marignani PA Woodgett JR Multiple phosphoinositide 3-kinase-dependent steps in activation of protein kinase B Mol Cell Biol 2002 22 6247 6260 10.1128/MCB.22.17.6247-6260.2002 12167717 PMC134003

[B35] Omar MA Wang L Clanachan AS Cardioprotection by GSK-3 inhibition: role of enhanced glycogen synthesis and attenuation of calcium overload Cardiovasc Res 2010 86 478 486 10.1093/cvr/cvp421 20053658

[B36] Lahair MM Howe CJ Rodriguez-Mora O McCubrey JA Franklin RA Molecular pathways leading to oxidative stress-induced phosphorylation of Akt Antioxid Redox Signal 2006 8 1749 1756 10.1089/ars.2006.8.1749 16987028

[B37] Lindvall J Islam TC Interaction of Btk and Akt in B cell signaling Biochem Biophys Res Commun 2002 293 1319 1326 10.1016/S0006-291X(02)00382-0 12054657

[B38] Cheung SMS Kornelson JC Al-Alwan M Marshall AJ Regulation of phosphoinositide 3-kinase signaling by oxidants: Hydrogen peroxide selectively enhances immunoreceptor-induced recruitment of phosphatidylinositol (3,4) bisphosphate-binding PH domain proteins Cell Signal 2007 19 902 912 10.1016/j.cellsig.2006.10.013 17215104

